# Robot-assisted partial knee replacement versus standard total knee replacement (RoboKnees): a protocol for a pilot randomized controlled trial

**DOI:** 10.1186/s40814-024-01463-x

**Published:** 2024-02-21

**Authors:** Kim Madden, Breanne Flood, Monica Malek, Vincent Milantoni, Janie L. Astephen Wilson, Jean-Eric Tarride, Vickas Khanna, Anthony Adili, on behalf of the RoboKnees Investigators, Kim Madden, Anthony Adili, Mohit Bhandari, Vickas Khanna, Jean-Eric Tarride, Lehana Thabane, Daniel Tushinski, Janie Wilson, Paul Zalzal, Breanne Flood, Monica Malek, Ashvin Moro, Kim Irish, Haider Khan, Vireshwar Jagdeo, Nathasha Rajapaksege, James Yan

**Affiliations:** 1 Department of Surgery, McMaster University, Hamilton, Canada; 2 Research Institute of St. Joseph’s Healthcare Hamilton, Hamilton, Canada; 3 Department of Health Research Methods, Evidence, and Impact, McMaster University, Hamilton, Canada; 4 School of Biomedical Engineering, McMaster University, Hamilton, Canada; 5 School of Biomedical Engineering, Dalhousie University, Halifax, Canada

**Keywords:** Robot-assisted surgery, Knee replacement, Randomized controlled trial, Protocol, Feasibility

## Abstract

**Background:**

Total knee arthroplasty is a common surgery for end-stage knee osteoarthritis. Partial knee arthroplasty is also a treatment option for patients with arthritis present in only one or two knee compartments. Partial knee arthroplasty can preserve the natural knee biomechanics, but these replacements may not last as long as total knee replacements. Robotic-assisted orthopedic techniques can help facilitate partial knee replacements, increasing accuracy and precision. This trial will investigate the feasibility and assess clinical outcomes for a larger definitive trial.

**Methods:**

This is a protocol for an ongoing parallel randomized pilot trial of 64 patients with uni- or bicompartmental knee arthritis. Patients are randomized to either receive robot-assisted partial knee arthroplasty or manual total knee arthroplasty. The primary outcome of this pilot is investigating the feasibility of a larger trial. Secondary (clinical) outcomes include joint awareness, return to activities, knee function, patient global impression of change, persistent post-surgical pain, re-operations, resource utilization and cost-effectiveness, health-related quality of life, radiographic alignment, knee kinematics during walking gait, and complications up to 24 months post-surgery.

**Discussion:**

The RoboKnees pilot study is the first step in determining the outcome of robot-assisted partial knee replacements. Conclusions from this study will be used to design future large-scale trials. This study will inform surgeons about the potential benefits of robot-assisted partial knee replacements.

**Trial registration:**

This study was prospectively registered on clinicaltrials.gov (identifier: NCT04378049) on 4 May 2020, before the first patient was randomized.

**Supplementary Information:**

The online version contains supplementary material available at 10.1186/s40814-024-01463-x.

## Background

Arthritis is a common set of joint diseases impacting 6 million Canadians. Osteoarthritis (OA) affects the cartilage, subchondral bone, and the surrounding musculature, tendons, and ligaments, leading to joint pain and reduced mobility [1]. There is no cure, but for severe OA, knee replacement surgery is an option. Over 70,000 knee replacements were performed in Canada in 2019–2020 [2] Approximately $1.4 billion (CAD) is spent on joint replacement surgeries annually [2]. However, one in five patients is dissatisfied with their new knee after surgery [3, 4].

Unicompartmental knee arthroplasty (UKA) conserves knee tissue in patients with OA in a single compartment, while total knee arthroplasty (TKA) removes at least one or both the anterior cruciate ligament (ACL) plus or minus the posterior cruciate ligament (PCL), causing a loss of proprioception, knee stability, which can result in a change in natural knee kinematics [5]. Bicompartmental knee arthroplasty (BiKA) is an option for patients with OA in two compartments. Partial knee replacement may be suitable for as many as one-quarter to one-half of knee replacement candidates [6], but more than 90% receive TKA [7], suggesting underutilization of partial knee replacements [6–8]. This may be in part due to the technical complexity and previously published increase in revision rates of manual partial knee replacements compared to total knee arthroplasty.

Numerous studies have been conducted comparing TKA and partial knee replacements [7–19]. The research has found that UKA can lead to improved functional outcomes, but with higher revision rates [9], although some patients report less pain [17] and greater satisfaction [20, 21]. Recent systematic reviews indicate that TKA generally provides better short-term outcomes, lower revision rates, and shorter operative times when compared to BiKA. However, BiKA may result in a slightly better post-operative range of motion (ROM) and less intraoperative blood loss [12, 15]. Despite the available research, it is still challenging to determine the advantages and disadvantages of partial knee arthroplasty due to the limited number of studies utilizing robot-assisted technology to perform partial knee replacements and inconsistent reported outcomes [10].

The use of surgeon-controlled robotic arm-assisted knee replacement is growing in popularity, but their effectiveness and cost-utility are unclear. The Mako RIO robotic arm, developed by Stryker, is a surgeon-controlled orthopedic robot that improves precision and accuracy in bone resections during arthroplasty surgeries. It employs advanced robotics and real-time tracking, providing haptic and visual feedback to guide surgeons in achieving precise movements and accurate bone resections. Additionally, the system uses CT-based imaging to create a virtual model of the patient’s anatomy, aiding in determining optimal implant placement. It is believed that by enhancing precision and repeatability, the Mako RIO robot enhances the overall outcomes of arthroplasty procedures [22].

Robotic technology can help facilitate partial knee replacements, as partial knee replacements are very technically challenging to do manually even for the most experienced surgeons. Research studies suggest that robotic technology significantly improves alignment, the precision of bone preparation and implant placement, and reduces technical variability and outliers [18], with some evidence supporting improved ROM and lower revision rates, but with increased surgery time and cost [23]. There is limited evidence on implant survival and long-term clinical outcomes [18]. One small cohort study evaluated 53 consecutive robot-assisted BiKA procedures at a single center with findings supporting successful survivorship at 5 and 7 years, predominantly excellent postoperative functional outcomes, and overall good patient satisfaction [24]. An additional single-surgeon study retrospectively examined 1018 knees of patients who underwent robot-assisted UKA, patellofemoral arthroplasty, or BiKA. Findings included a robust 5-year survivorship, favorable KOOS scores, and high satisfaction. The study implies that robot-assisted knee surgery may offer enhanced lower limb alignment, improved component positioning, and precise ligament balance, potentially justifying the additional costs through improved outcomes and reduced healthcare expenses [25]. However, more high-quality comparative research is needed to draw conclusions about the benefits of robotic technology in knee replacements.

## Objectives

The primary objective of this trial is to assess the feasibility of conducting a larger definitive trial to assess robot-assisted partial knee replacements versus manual TKA (standard care) on functional outcomes, recovery, and patient-reported outcomes. Specific feasibility objectives include assessing our participant recruitment and retention plan, identifying and resolving any problems with data quality, and identifying and resolving any problems with implementing the study treatment (e.g., crossovers, expertise, technological and process issues).

The clinical objectives of this pilot trial will be the objectives of the definitive trial. These objectives are exploratory only in this pilot trial. The clinical objectives of this pilot trial are to establish preliminary estimates of the effects of robot-assisted partial knee replacements versus manual TKA on the following outcomes: joint awareness, return to activities (e.g. work, leisure, domestic duties), knee function, persistent post-surgical pain, patient global impression of change, re-operations/implant survival, health-related quality of life (HRQoL), resource utilization and cost-effectiveness, radiographic alignment, knee kinematics during walking gait, and safety.

## Methods

This trial was registered with clinicaltrials.gov prior to any patient enrollment (NCT04378049). This protocol is reported according to Standard Protocol Items: Recommendations for Interventional Trials (SPIRIT) reporting guidelines [26] and the pilot results paper will follow the pilot study extension to the Consolidated Standards of Reporting Trials (CONSORT) guidelines [27].

### Study design

**Fig. 1:**
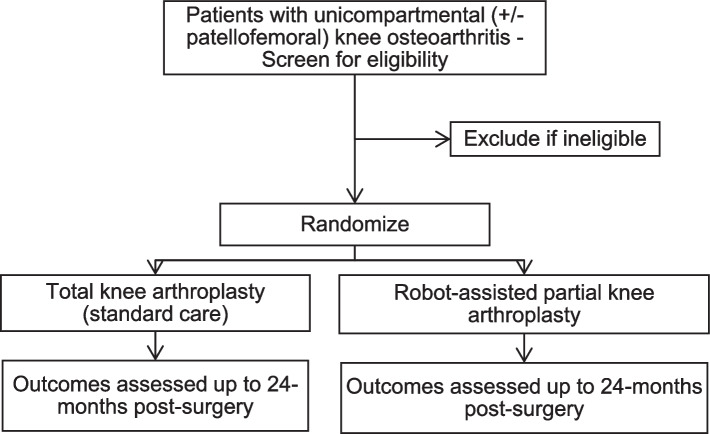
Schematic of study design

We are conducting a pilot 2-group parallel randomized controlled trial (RCT) assessing the feasibility of conducting a large definitive trial of robot-assisted partial knee arthroplasty versus TKA (standard care) on functional outcomes, recovery, and patient satisfaction. Eligible patients presenting with uni- or bicompartmental compartmental knee osteoarthritis will be randomized to receive one of the two study interventions. A schematic diagram of the study design is presented in Fig. 1.

### Rationale for a pilot trial

A pilot trial is required prior to a larger definitive trial to ensure the larger trial’s methodological design and practical execution. The lessons learned in this pilot trial will be used to make necessary adjustments to the study design and procedures for use in a definitive trial. If appropriate (e.g., no major methodological changes), patients from the pilot study will be included in the definitive trial for efficiency.

### Study setting

As of the date of this protocol, St. Joseph’s is one of two Canadian institutions that currently has access to an orthopedic surgical robot. St. Joseph’s is an academic hospital that receives referrals from the Musculoskeletal Central Intake and Assessment Centre serving much of southwestern Ontario including residents in Hamilton, Niagara, Haldimand, and Brant. Surgeons at St. Joseph’s conducted approximately 400 knee replacements in 2022, and plan to increase the number of knee surgeries to 750 per year in 2023 by utilizing new regional rooms.

### Eligibility criteria

We selected broad eligibility criteria to increase the generalizability and pragmatism of the trial.

### Inclusion criteria

Eligible patients must meet all of the following inclusion criteria to be considered for the trial;

1. Adult (18+).
2. Uni- or bicompartmental knee OA, requiring surgical treatment.
3. Two study surgeons independently agree that the patient is eligible for either treatment group.

### Exclusion criteria

Patients will be excluded for any one or more of the following reasons;

1. Inability to provide informed consent (e.g., cognitive disability, language barrier).
2. Revision knee surgery.
3. Simultaneous bilateral knee surgery.
4. Previous major knee surgery or trauma.
5. The robot or required components are unavailable (e.g., technical difficulties, robot-specific disposables out of stock).
6. A CT scan cannot be obtained prior to surgery.
7. Patient does not wish to participate.
8. Any known factor, disease, or clinically relevant medical or surgical conditions that, in the opinion of the investigator, might put the subject at risk, interfere with treatment compliance, study conduct, or interpretation of the results.

### Screening and recruitment

Enrolling surgeons will identify potentially eligible patients from their clinics around the time that surgery is scheduled. The research coordinator will explain the trial in further detail, confirm eligibility, and obtain informed consent. We will obtain a CT scan for all participants prior to surgery.

### Intervention group

Participants with isolated medial or isolated lateral compartment OA who are randomized to the intervention group and have one affected knee compartment will receive a robot-assisted UKA. If the patient has medial or lateral OA plus patellofemoral OA they will receive a BiKA consisting of two simultaneous UKAs. The partial knee replacement procedures will be performed using the Mako RIO robotic arm (Stryker) according to the manufacturer’s instructions. The study interventions are all approved by Health Canada for the study indications. The choice of implant and use of bone cement will be recorded but will be left to the surgeon’s discretion. Surgeons will resurface the patella if patients have a positive preoperative grind test and patellar cartilage meets Outerbridge grade 3 or 4 criteria [28].

### Control group

Participants who are randomized to the control group will undergo TKA according to the local standard of care. The choice of implant and use of bone cement will be recorded but left to the surgeon’s discretion according to their standard practice.

### Minimizing expertise bias

All study surgeons are high-volume expert TKA surgeons, but robot-assisted partial knee replacements are new procedures in Canada. Our research group received an education grant from McMaster Surgical Associates that has allowed our surgeons to gain experience in robot-assisted TKA, UKA, and BiKA before the trial began. Previous research on robot-assisted UKA learning curves shows that high-volume arthroplasty surgeons can accurately place the implants starting from the first case, and that operative time and anxiety levels drop substantially after 6 cases [29]. Therefore, each surgeon must complete the Mako RIO certification course (Stryker), then train on 6 cases each with a senior surgeon before completing study surgeries. Over the course of these 6 expertise-building cases, the surgeons must demonstrate that they can confidently perform the steps in the robot-assisted procedure, their surgical time must drop to typical lengths, their post-op alignment must be within an acceptable range, and the patients must be free of major postoperative complications attributable to the robot-assisted procedure. Each study surgeon must meet these expectations before enrolling patients into this trial to minimize the risk of expertise bias.

### Adherence and crossovers

As part of the feasibility objectives for this pilot trial, we will closely monitor treatment adherence and crossovers, and we will identify any unanticipated problems prior to the definitive trial. There will typically be opportunities to correct any technological issues that arise prior to the case. We will work closely with Diagnostic Imaging, the Mako Product Specialist, and operating room managers to ensure the availability of all robot-specific imaging and disposable supplies, and to ensure the smooth operation of the robotic technology.

### Contamination and co-interventions

Peri-operative and post-operative care, pain management, and physiotherapy have been standardized across our local health administration region according to current evidence-based best practices. All enrolling surgeons adhere to these standard practices.

### Randomization and allocation concealment

Patients will be randomized 1:1 to either the treatment or control group using a centralized online randomization system to ensure allocation concealment. A statistician who is otherwise not involved in the trial will prepare the randomization sequence and program the online randomization system. Research coordinators or enrolling surgeons will randomize eligible and consenting participants on the date of surgery to minimize the risk of bias.

### Blinding

Surgeons cannot be blinded to the treatment group. It would not be feasible to blind the health care team and patients to total versus partial replacement because the incisions and imaging are visually distinguishable. We will attempt to minimize bias in subjective measures by presenting patients with balanced information on each treatment group prior to the trial. Patients will be blinded to whether they are receiving manual or robot-assisted treatment to minimize bias associated with expectations regarding robot-assisted surgery. Although all participants who receive partial knee replacements will receive robot-assisted surgery (i.e., this is a 2-group trial, not a 2 × 2 factorial trial), we will inform patients that they will have an equal chance of being in the partial or total replacement group and an equal chance of being in the robot-assisted or manual surgery group. All participants will receive a CT scan prior to surgery to maintain blinding to treatment assignment. Data analysts will be blinded to treatment allocation, and outcome assessors will be blinded wherever possible. Aggregate-level data will be blinded at all times except to the Independent Medical Monitor for safety monitoring purposes. An independent outcomes assessor will independently review the radiographic alignment for each patient and review the relatedness of adverse events and re-operations. The independent assessor cannot be blinded because imaging is visually distinguishable.

### Outcome measures

#### Primary outcome: feasibility

The primary outcome of this trial is feasibility. Specifically, feasibility outcomes include: assessing our participant recruitment and retention plan based on whether the recruitment target is reached in a timely manner, identifying and resolving any problems with data quality through achieving a follow-up rate of 90% and questionnaire completion exceeding 80%, and identifying and resolving any problems with implementing the study treatment (e.g., crossovers, expertise, technological issues) this includes less than 5% crossovers.

#### Secondary outcomes: clinical outcomes

The clinical outcomes of this pilot trial are meant to establish preliminary estimates of the effects of robot-assisted partial knee replacement vs. standard TKA. Specific clinical outcomes and how they will be measured are listed below. These will be the outcomes of the definitive trial.

### Return to activities

We will use the Return To Function (RTF) questionnaire developed by Busse et al. at McMaster University. The RTF questionnaire is a 5-item questionnaire that has been used previously in orthopedic trials, including an FDA-regulated orthopedic device trial [30], to determine when a trial participant returns to work, leisure, and activities around the home after an injury or surgery.

### Joint awareness

We will use the Forgotten Joint Score (FJS-Knee) to measure joint awareness. The FJS-Knee is a 12-item questionnaire that aims to assess both function and feeling by asking about patients’ awareness of their artificial knee while doing various daily activities. For example, “Are you aware of your artificial joint when you are in bed at night?” Joint awareness can help distinguish which patients are satisfied with their joint replacement versus those who are unsatisfied, by comparing it to a natural-feeling knee [31].

### Knee function

We will measure knee function using the Oxford Knee Score (OKS) and range of motion (ROM). The OKS is a well-validated and well-used 12-item questionnaire specifically used to measure knee function when performing daily activities after total knee replacement surgery [32, 33]. We will measure knee ROM using our motion capture gait lab at in-clinic visits.

### Patient global impression of change

The Patient Global Impression of Change (PGIC) scale is widely used in pain research to assess patients’ beliefs about the efficacy of treatment. The PGIC is one item and asks patients to rate the effectiveness of the treatment of interest on a 7-point ordinal scale ranging from “very much improved” to “very much worse” [34].

### Persistent post-surgical pain (PPSP)

We will use a modified version of the WHO’s definition of PPSP [35] to include a minimum threshold of pain severity:

1. Pain that began after surgery or a tissue trauma;
2. Pain is in an area of preceding surgery or tissue trauma;
3. Pain has persisted for at least 3 months after surgery; and
4. The pain is not better explained by an infection, malignancy, a pre-existing pain condition or any other alternative cause.
5. ≥ 4 out of 10 on the numeric rating scale (NRS) from the Brief Pain Inventory (BPI-SF) for “average pain over the last week”. The scale ranges from 0 (no pain) to 10 (pain as bad as you can imagine) [36].

### Re-operations

We will report the number of revision surgeries within the study period including cases of periprosthetic joint infection, aseptic loosening, instability, polyethylene wear, intractable pain, periprosthetic fracture, etc. Re-operation will be defined as any surgery on the same joint requiring general or axial anesthesia; local procedures such as injections and local nerve blocks will not be included.

### Quality of life/health economics

We will use the Euro-Qol 5 Dimensions-5L (EQ-5D-5L) questionnaire to assess health-related quality of life (HRQoL). The EQ-5D-5L is a well-validated and well-used quality-of-life instrument that can assess health utilities for the purpose of health economic analyses (e.g. cost-effectiveness) [37]. We will also collect healthcare resource utilization information (e.g., hospitalization, physician visits, physiotherapy) and information on productivity (e.g., time missed from work) to assist with health economic analyses.

### Radiographic alignment

We will measure mechanical alignment on pre-operative and post-operative weight-bearing long-leg X-rays. Specifically, we will measure coronal plane mechanical axis measurements using the angle between the mechanical femoral axis and the mechanical axis of the tibia.

### Knee kinematics

Overground, instrumented walking kinematic gait analysis will be performed in the clinic environment at four time points (pre-operatively within 1 month of surgery, 3, 6, and 12 months post-surgery) using a 14-camera optoelectronic motion capture system (Optitrack, NaturalPoint, Corvallis, OR, USA) installed on raised mounts in a clinic hallway. Reflective markers will be placed in strategic anatomical locations on the lower extremities of patients for each testing session. A custom marker placement protocol that includes multiple triads of markers on each limb in addition to anatomical locations will be used for redundancy of limb segment pose estimation given the constrained camera viewing volume of the hallway. After system calibration and standing static calibration, patients will be asked to walk at their comfortable self-selected walking speed down the instrumented hallway. Five to seven steady-state speed gait trials will be recorded with positional information recorded at 120 Hz. Total gait testing time will not exceed 15 min. Stride characteristics (walking velocity, stride length, step length, step width) and three-dimensional knee, hip, and ankle angles through the gait cycle will be modeled using Visual 3D software (C-Motion Inc.). Kinematic modeling and marker set-up will follow previously published work for a similar clinical population by co-applicant Wilson et al. [38]. Primary gait outcomes will include the knee joint flexion and adduction angle magnitudes and range during the stance and swing phase of gait. Secondary gait outcomes will include stride characteristics as defined above, knee transverse rotation during stance, hip and ankle range of motion, and peak magnitudes during stance. For patients completing an optional inertial sensor measurement unit (IMU) collection, outcomes will include spatiotemporal parameters (stance time, swing time, step length) and impact accelerations from the wearable Axivity AX6 (100 Hz sampling, Hz, Axivity Ltd., Newcastle, UK) sensors. For additional information see Appendix 2.

### Safety: surgery-related adverse events

We will collect all serious adverse events (AEs) and surgery-related non-serious AEs throughout the trial for safety monitoring purposes.

### Participant follow-up

We will follow participants for 24 months after surgery. We will collect baseline data at the standard pre-operative clinical visit or the pre-operative education session. Follow-up visits will be at 6 weeks, 3 months, 6 months, 12 months, and 24 months after surgery. Visits at 24 months may be completed by telephone if the participant does not wish to come to the hospital. See Table 1 for a summary of study events and measurements at each time point.

**Table 1:** Study visit summary

Study visit	Pre-op	6 weeks post-op	3 months post-op	6 months post-op	12 months post-op	24 months post-op
Visit window	1–4 weeks pre-op	5–7 weeks post-op	2–4 months post-op	5–7 months post-op	11–13 months post-op	22–26 months post-op
Visit type	In-person	In-person	In-person	In-person	In-person	In-person or telephone
Screening	X					
Consent	X					
Randomization	X					
Demographics and baseline info	X					
CT scan	X					
FJS-Knee		X	X	X	X	X
RTF		X	X	X	X	X
OKS	X	X	X	X	X	X
ROM	X	X				
PGIC		X	X	X	X	X
PPSP				X	X	X
Re-ops		X	X	X	X	X
EQ-5D	X	X	X	X	X	X
Healthcare use		X	X	X	X	X
Alignment	X	X			X	X
Gait	X		X	X	X	
AEs		X	X	X	X	X

*CT* computed tomography, *FJS* Forgotten Joint Score, *RTF* Return To Function Questionnaire, *OKS* Oxford Knee Score, *ROM* range of motion, *PGIC* Patient Global Impression of Change Scale, *PPSP* persistent post-surgical pain, *Re-ops* re-operations, *EQ-5D* EuroQol 5 Dimensions, *Alignment* radiographic alignment, *AEs* adverse events

### Participant retention

Once a participant is enrolled in the trial, every reasonable effort will be made to follow the participant for the entire duration of the study period. Previously established orthopedic-specific procedures developed and refined by our team will be implemented to improve participant retention [39]. Participants may decide to withdraw from this trial at any time. If a participant withdraws prior to completing the trial, research personnel will document the reason for withdrawal and attempt to collect any available outcome data (e.g., at least vital status and safety). Participants will not be withdrawn from the study due to lack of adherence to the study protocol (e.g., participant received wrong intervention, missed follow-up visits).

### Sample size consideration

Our sample size is based on the confidence interval around the percentage of complete follow-up (feasibility objective) using the method for calculating sample size in pilot trials suggested by Thabane et al. [40]. We will aim for 90% follow-up but we will consider the trial successful if we achieve at least 80% follow-up for our primary clinical outcome. Therefore, to achieve a margin of error of 10%, we will require 64 participants (32 per group). If at least 58/64 (90.6%) of participants achieve successful follow-up, the lower boundary of the 95% confidence interval will be above 80% and we will consider the trial feasible. We may use data from this pilot trial to inform the definitive trial sample size calculation.

### Statistical methods

The analysis and reporting of results will follow the CONSORT guidelines for reporting randomized pilot and feasibility trials [41]. We will use an intention-to-treat analysis. The process of participant enrollment and flow throughout the study will be summarized using a CONSORT flow diagram. The clinical outcomes of this pilot trial are meant to be exploratory only, as the pilot trial is not powered for definitive analyses. Our feasibility analysis will be descriptive and will include point estimates with 95% confidence intervals. Planned exploratory analysis of clinical (secondary) objectives is summarized in Appendix Table 1.

### Steering committee

Trial conduct will be overseen by a steering committee comprised of a team including orthopedic surgeons, engineers, gait analysis experts, orthopedic research methodologists, biostatisticians, and health economists.

### Independent medical monitor

We will not require a full Data and Safety Monitoring Committee (DSMC) for this pilot trial given that all devices and equipment are approved by Health Canada for the indications described in this protocol, and all procedures are in use in standard practice. An Independent Medical Monitor will annually review information on revisions, adverse events, and serious adverse events in an unblinded manner. Any concerning trends in the rate or pattern of AEs will be communicated to the steering committee. Unanticipated problems resulting in risk to participants or others will be reported within 24 h of becoming aware of the problem. No formal interim analysis will be necessary for this pilot RCT.

### Data management

Participant data will be collected and recorded on study-specific case report forms and Research personnel at the clinical site will submit the required data, as detailed on the case report forms, to the methods centre using the REDCap electronic data capture system. Only research personnel with appropriate training will have access to the data.

### Confidentiality

To safeguard confidentiality before, during, and after the trial, personal information about participants will be collected, shared, and maintained using secure methods in accordance with data protection protocols.

### Ethics and dissemination

The Hamilton Integrated Research Ethics Board (HiREB) provided approval prior to initiating this trial protocol (project #10961). Participants will provide written informed consent before participating in the study.

Any modifications to the study protocol that could impact the study’s implementation, participants’ safety, or potential benefits (such as alterations to study objectives, design, sample size, or procedures) will necessitate a formal amendment to the protocol. These amendments will be subject to approval by the Principal Investigator and the ethics board. Administrative adjustments (such as minor corrections or clarifications that do not alter the study’s conduct) will not require a formal amendment process.

All results from the study will be submitted for publication regardless of the findings with attempts to ensure the amount of time between completion of data collection and release of study findings is minimized. We intend to make the results available in a peer-reviewed journal, at conferences and will publish results of pre-registered outcomes on the clinicaltrials.gov registry. We will engage our institution’s press offices to disseminate the results to the general public and use formal and informal social media channels for broader uptake. The results of this pilot study will also directly inform the definitive study and future directions for research in this area.

### Supplementary Information

**Additional file 1: **Appendix Table 1: Planned summary of feasibility and clinical objectives, outcome measures and exploratory analysis

**Additional file 2:** Appendix 2: Optional Sensor Data Collection

**Additional file 3.**

## Data Availability

Not applicable.

## Abbreviations

ACL: Anterior cruciate ligament
BiKA: Bicompartmental knee arthroplasty
BPI-SF: Brief Pain Inventory
EQ-5D-5L: EuroQol-5 Dimensions
FDA: U.S. Food and Drug Administration (FDA)
FJS: Forgotten Joint Score
HRQoL: Health-related quality of life
NRS: Numeric rating scale
OA: Osteoarthritis
OKS: Oxford Knee Score
PCL: Posterior cruciate ligament
PGIC: Patient Global Impression of Change
PPSP: Persistent post-surgical pain
RCT: Randomized controlled trial
ROM: Range of motion
RTF: Return to Function questionnaire
TKA: Total knee arthroplasty
UKA: Unicompartmental knee arthroplasty
WHO: World Health Organization
