# Supplementary material for: Robot-assisted partial knee replacement versus standard total knee replacement (RoboKnees): a protocol for a pilot randomized controlled trial
Source: Pilot Feasibility Stud. 2024 Feb 21;10:39. doi: 10.1186/s40814-024-01463-x (PMC10880336; doi:10.1186/s40814-024-01463-x)
Supplement: Supplementary file 1 — Additional file 1: Appendix Table 1: Planned summary of feasibility and clinical objectives, outcome measures and exploratory analysis [file 40814_2024_1463_MOESM1_ESM.docx]

**Appendix Table 1**

| Planned summary of feasibility and clinical objectives, outcome measures and exploratory analysis | | | |
| --- | --- | --- | --- |
| **Objective** | **Type of outcome** | **Outcome measure** | **Method of Analysis** |
| Rate of participant recruitment | Feasibility | Number of participants recruited over time | Descriptive |
| Participant retention | Feasibility | Number of participants who complete follow-up at 24 months post-op | Descriptive |
| Adherence to protocol | Feasibility | Number of patient crossovers | Descriptive |
|  |  | Number of randomization errors | Descriptive |
| Level of data quality | Feasibility | Completeness of data | Descriptive |
| Assess recovery | Clinical | RTF questionnaire | Descriptive - categories  Linear regression – percent of function regained |
| Assess knee function | Clinical | FJS | Linear regression |
|  |  | OKS | Linear regression adjusted for baseline OKS |
|  |  | ROM | Linear regression adjusted for baseline ROM |
| Assess patient treatment satisfaction | Clinical | Patient global impression of change scale | Linear regression |
| Assess pain | Clinical | WHO assessment of PPSP | Logistic regression |
| Assess short-term implant survival | Clinical | Implants not requiring revision surgery | K-M survival, Cox PH regression |
| Assess health-related quality of life | Clinical | Quality Adjusted Life years (i.e. based on EQ-5D-5L health utilities) | GLM adjusted for baseline utility |
| Assess health care resource utilization, productivity gain/losses and associates costs | Economics | Direct healthcare costs  (costed using publicly  available sources) | Count data models for  healthcare resource utilization  and days missed from work  and GLM for costs |
| Assess radiographic alignment | Clinical | Degrees of mechanical malalignment from x-rays | Linear regression; adjusted for baseline malalignment |
| Assess gait mechanics | Clinical | Knee flexion and adduction angles and range of motion during stance, spatiotemporal parameters, and impact accelerations | Generalized linear model comparing change values from pre-operative to 3-, 6- and 12-month states, controlling for gait velocity |
| Assess peri-operative and post-operative complications | Clinical | Number of patients with complications | Descriptive |
| *K-M – Kaplan-Meier; Cox PH – Cox proportional hazards; EQ-5D – EuroQol 5 Dimensions; FJS – Forgotten Joint Score; RTF – Return To Function Questionnaire; OKS – Oxford Knee Score; PPSP – persistent post-surgical pain; GLM – generalized linear model; ROM – Range of motion; WHO – World Health Organization* | | | |
