# Supplementary material for: Robot-assisted partial knee replacement versus standard total knee replacement (RoboKnees): a protocol for a pilot randomized controlled trial
Source: Pilot Feasibility Stud. 2024 Feb 21;10:39. doi: 10.1186/s40814-024-01463-x (PMC10880336; doi:10.1186/s40814-024-01463-x)
Supplement: Supplementary file 2 — Additional file 2: Appendix 2: Optional Sensor Data Collection [file 40814_2024_1463_MOESM2_ESM.docx]

**Appendix 2 – Optional Sensor Data Collection**

For the optional sensor data collection, two wearable sensors (Axivity AX6) will be attached to the patients’ knees with medical grade tape and record movement outside of the lab environment for no longer than 1 week at 100 Hz. The sensors are placed on each leg at the anterior-medial aspect of the proximal tibia using medical grade adhesive tape. During this period, patients were instructed to go about their normal daily activities. At the end of the testing period, the patient will mail the sensors back in an envelope provided by the researchers. Upon return the data will be segmented into walking periods and non-walking periods. The walking periods will be analyzed for stride characteristics and peak values in each plane of motion during stance for each step.
